# Interaction of the Blood Components with Ascending Thoracic Aortic Aneurysm Wall: Biomechanical and Fluid Analyses

**DOI:** 10.3390/life12091296

**Published:** 2022-08-24

**Authors:** Ramezan Ali Taheri, Reza Razaghi, Ali Bahramifar, Mahdi Morshedi, Majid Mafi, Alireza Karimi

**Affiliations:** 1 Nanobiotechnology Research Center, Baqiyatallah University of Medical Sciences, Tehran 1435916471, Iran; 2 Department of Ophthalmology and Visual Sciences, University of Alabama at Birmingham, Birmingham, AL 35233, USA; 3 Trauma Research Center, Baqiyatallah University of Medical Sciences, Tehran 1435916471, Iran; 4 Department of Surgery, Trauma Research Center, Baqiyatallah University of Medical Sciences, Tehran 1435916471, Iran; 5 Biomedical Engineering Research Center, Baqiyatallah University of Medical Sciences, Tehran 1435916471, Iran

**Keywords:** ascending thoracic aortic aneurysm, red blood cell, white blood cell, plasma, discrete element, fluid–structure interaction, computational fluid dynamics

## Abstract

Background: Ascending thoracic aortic aneurysm (ATAA) is an asymptomatic localized dilation of the aorta that is prone to rupture with a high rate of mortality. While diameter is the main risk factor for rupture assessment, it has been shown that the peak wall stress from finite element (FE) simulations may contribute to refinement of clinical decisions. In FE simulations, the intraluminal boundary condition is a single-phase blood flow that interacts with the thoracic aorta (TA). However, the blood is consisted of red blood cells (RBCs), white blood cells (WBCs), and plasma that interacts with the TA wall, so it may affect the resultant stresses and strains in the TA, as well as hemodynamics of the blood. Methods: In this study, discrete elements were distributed in the TA lumen to represent the blood components and mechanically coupled using fluid–structure interaction (FSI). Healthy and aneurysmal human TA tissues were subjected to axial and circumferential tensile loadings, and the hyperelastic mechanical properties were assigned to the TA and ATAA FE models. Results: The ATAA showed larger tensile and shear stresses but smaller fluid velocity compared to the ATA. The blood components experienced smaller shear stress in interaction with the ATAA wall compared to TA. The computational fluid dynamics showed smaller blood velocity and wall shear stress compared to the FSI. Conclusions: This study is a first proof of concept, and future investigations will aim at validating the novel methodology to derive a more reliable ATAA rupture risk assessment considering the interaction of the blood components with the TA wall.

## 1. Introduction

Ascending thoracic aortic aneurysm (ATAA) is a degenerative disease [1] and the 12th leading cause of death in the United States [2]. Aneurysm formation is associated with wall remodeling, radial enlargement, alteration in the blood hemodynamics, and rupture [3]. Aortic rupture typically initiates by a tear in the intima layer [4], causing an in-plane over-stress in the media layer, exceeding its wall strength, and, finally, propagation to the inner media before delamination [5]. While degeneration of elastin fibers due to aneurysm growth is also responsible for weakening of the aortic wall [6,7], understanding the biomechanics of the thoracic aortic aneurysms (TAAs) and hemodynamics of the blood may significantly contribute to clinical diagnosis and treatment [8,9].

While it has been suggested that a vessel with a diameter higher than 1.5x may be considered as an indicator for aneurysm diagnosis [10], recent studies revealed that the diameter is perhaps not sufficient to evaluate the risk of rupture [11] since large ATAAs might be asymptomatic [12]. The peak wall stress derived from numerical simulations, i.e., finite element (FE), can relevantly complete the current diameter diagnosis approach [11]. Computational models based on the FE method can identify rapidly growing TAAs [13], assess rupture risk [14], and, in some cases, may even contribute to the prediction of the rupture site [15].

The biomechanical factors that may influence the stresses in TAs are mechanical properties and geometry [16,17]. As developing an aneurysm is associated with growing stiffness in the TA tissue [18,19], characterization of the biomechanical properties of the aortic wall would significantly contribute to early diagnosis of an aneurysm before rupture occurs. Various mechanical parameters, such as tensile strength, initial elastic modulus, maximum elastic modulus, as well as failure or yield stresses and strains, have been reported for healthy TAs and TAAs [20,21,22,23,24]. ATAs and DTAs have different wall structures due to the regional variation in the distribution and structure of elastin and collagen fibers from the proximal to the distal end of the aorta [25,26]. Iliopoulos et al. showed that the elastin content is significantly decreased in aneurysms, causing a smaller failure strain, with higher maximum elastic modulus but nearly equal failure stress compared to healthy TAs [26]. A robust and reliable estimation of aneurysm rupture requires a patient-specific biomechanical model of the aorta [27]. Trabelsi et al. employed a patient-specific model of the aorta to compute the stresses of ATAAs on the basis of three rupture risk criteria, i.e., the maximum diameter, rupture risk index, and overpressure [28]. Suito et al. used fluid–structure interaction (FSI) simulations to analyze the blood flow in the TAs [29]. Yeh and his colleagues also used FSI to evaluate the hemodynamics in ATAAs between normal and hypertensive cases [30]. ATAA development and progression change the hemodynamics of the blood in the aorta [31]. However, the blood is consisted of red blood cells (RBCs) and white blood cells (WBCs) that are floating in the plasma with their own molecular weights, constituting ~55% of the total blood volume [32]. While RBCs can bear large deformations under external forces [33], WBCs are not as compliant as RBCs [34,35]. Karimi and colleagues used a fully mesh-free particle approach, namely smoothed particle hydrodynamics, to model the blood as a fluid with free surfaces with the RBC, WBC, and plasma [36]. While great strides have been made in modeling the ATAAs in humans using various numerical techniques, gaps in our knowledge of interaction between the RBCs, WBCs, and plasma with the ATAA wall and its contribution to the hemodynamics of the blood remain. Simulation of blood flow in TAs using discrete elements via a particle-based fully Lagrangian mesh-free method would significantly help to address the biomechanics of the ATAA in interaction with blood components. While hemodynamics properties cannot be directly linked to the risk of rupture of aneurysms [37], the resultant stresses and strains in the endothelium through the wall shear stress may contribute to weakening the aortic wall [38]. However, the contribution of the wall shear stress in the progression of ATAAs and the biochemical and biomechanical interaction with the intramural processes are largely unknown.

In this study, FE models of the TA and ATAA were reconstructed. The mechanical properties of the healthy and diseased TAs in *n* = 9 healthy and *n* = 9 aneurysmal cadavers were measured through axial and circumferential tests. The nonlinear hyperelastic (five-parameter Mooney–Rivlin) material model was used to address the mechanical properties of the TA and ATAA tissues. The blood components, such as RBC, WBC, and plasma, were distributed in the TA and ATAA lumen and mechanically coupled with the blood through the FSI method. The resultant stresses and strains in the TA and ATAA walls, as well as the hemodynamics of the blood, were calculated and compared. Computational fluid dynamics (CFD) was also used to calculate the velocity and wall shear stress of the blood in both the healthy and diseased TA.

## 2. Materials and Methods

### 2.1. Human Donors and Mechanical Measurements

**Figure 1:**
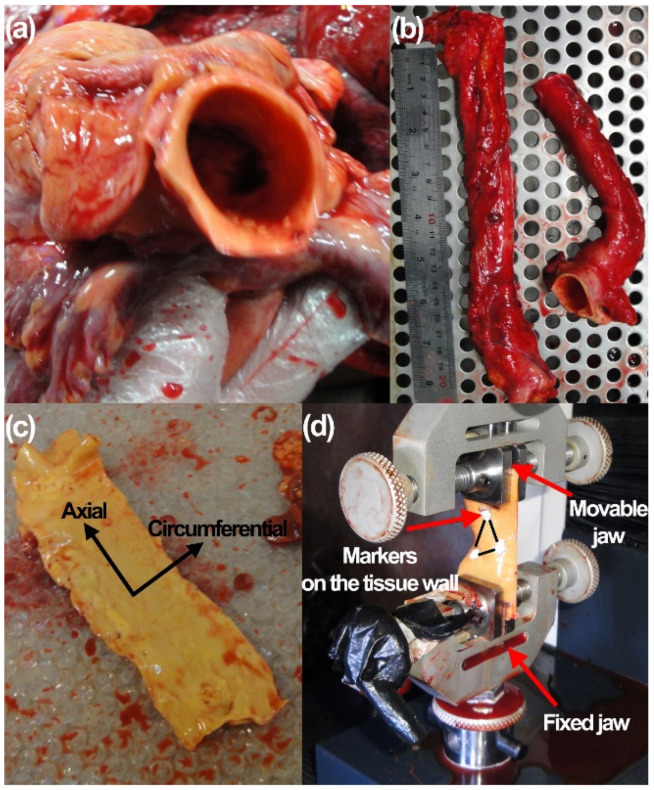
(**a**) Aortas from human cadavers. (**b**) ATAs, ATAAs, and DTAs were cut separately and (**c**) prepared for the axial and circumferential tensile tests. (**d**) Tissues were then mounted on the testing machine and three markers were attached on the tissue for digital image correlation measurements.

Thoracic aorta of donors from the left ventricle to the abdomen during autopsy of *n* = 9 healthy and *n* = 9 aneurysmal male individuals aged 56 ± 10 and 59 ± 9 year-old (mean ± SD), respectively, were collected. All procedures were carried out following agreement of the institutional review board of Basir Hospital, Tehran, Iran based on 2008 Declaration of Helsinki. Informed consent was obtained from the family of all donors. The aortic tissues were collected as fresh as possible within 5-h post-mortem, as shown in Figure 1a. Tissues around the aorta were removed carefully using a surgical scalpel, as shown in Figure 1b.

The luminal diameter and the thickness of the prepared tissues in unpressurized configuration were measured using a high precision digital caliper (Insize, Munich, Germany). For each specimen, the thickness was measured at 10 different cross-sections and then averaged. The ATAs, DTAs, and ATAAs were carefully cut for the mechanical measurement along the axial and circumferential directions, as indicated in Figure 1c. To reduce the degradation of the tissue samples after removal from the cadavers, TA samples once harvested were stored in a solution of 0.90% *w*/*v* of Dulbecco phosphate buffered saline without calcium and magnesium under 4–5 °C [39,40]. The tissue samples were removed from the solution and mounted on a uniaxial testing machine (Insize, Vienna, Austria) as illustrated in Figure 1d. To ensure a suitable humidity condition for the tissues, physiological solution was constantly sprayed on the tissues. To minimize the slippage effect between the tissues and jaws of the machine, a pair of coarse sandpapers were glued to the lower and upper jaws of the machine. The testing machine was equipped with a 50 kgf load cell (DBBP-50, Bongshin Company, Seongnam, Korea). All tests were carried out at room temperature (22 °C) and humidity of 52% (AcuRite, Lake Geneva, WI, USA). Each specimen was preconditioned with 10 cycles at a constant crosshead speed of 5 mm/min to diminish the effect of stress relaxation in the mechanical response of the tissues (data are not reported here) and then axially and circumferentially loaded with the constant crosshead speed of 5 mm/min until failure [41,42]. To enhance the accuracy of deformation/strain measurement in the tissue samples, the digital image correlation (DIC) technique was employed through bright triangle markers on the tissues, as shown in Figure 1d.

### 2.2. Statistical Analysis

Data from the axial and circumferential tensile experiments of *n = 9* healthy and *n = 9* aneurysmal thoracic aortas were determined to be normally distributed. The statistical significance of the difference between sample means was evaluated using a randomized one-way analysis of variance (ANOVA). When indicated by a significant F statistic after a one-way ANOVA, post hoc comparisons with the Scheffe method [43] were used to determine the individual levels of significant differences among the material parameters for the healthy and diseased TAs. The criterion chosen to discard the null hypothesis was *p* < 0.05.

### 2.3. Finite Element Model of the Aortic Wall

**Figure 2:**
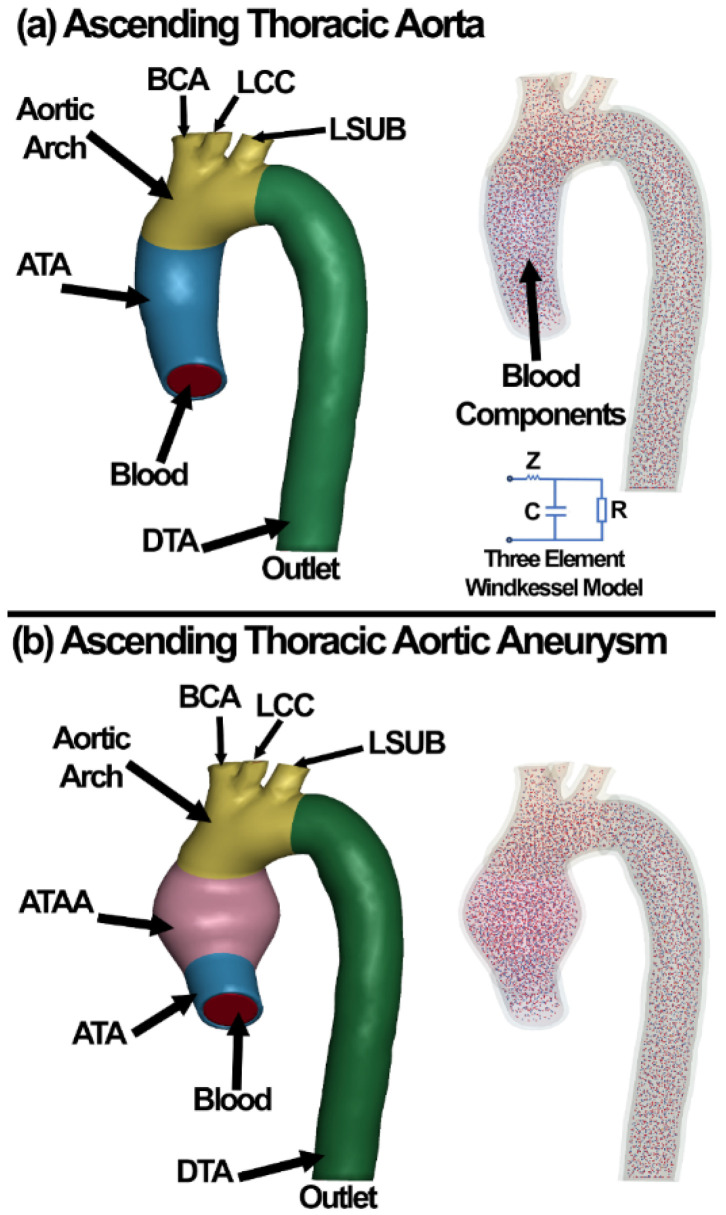
FE models of the (**a**) ATA and (**b**) ATAA. The blood components, i.e., RBCs, WBCs, and plasma, are distributed in the lumen. Three-element Windkessel model was defined in the outlet of both the ATA and ATAA FE models.

The geometrical models were established based on high-resolution contrast-enhanced magnetic resonance imaging with a slice thickness of 1.0 mm and pixel size of 0.625 mm (Activion, Toshiba Medical Systems Corporation, Tokyo, Japan) with an XY-resolution of 512 × 512 pixels. The images were segmented using Mimics (Materialise NV, Leuven, Belgium) with a suitable threshold filtration to create a surface mesh for a healthy ATA. The ATA geometry was deformed to create a generic ballooned shape to represent the ATAA. Early studies of ATAAs often assumed a uniform wall thickness from the distal up to the proximal sites [8]; however, the wall thickness value is known to have a significant influence in the biomechanical analyses [44,45]. Thus, based on our in vitro measurements for the ATA, DTA, and ATAA, we assigned a thickness varying between 2.31 (thickest) and 1.91 mm (thinnest) from proximal to distal positions, as summarized in Table 1. We also pre-loaded the model to ~100 mmHg (80–120 mmHg normal blood pressure range), and then the model was subjected to the load boundary. The TA and ATAA surface meshes were volume-meshed using our recently developed meshing algorithm [46,47]. The models meshed with 10-noded tetrahedral elements, as shown in Figure 2 (smooth mesh). To make sure that the FE results are both mesh- and time-independent, time-averaged 1st principal stress values in the aorta wall were compared for a model with five different element edge lengths. A time step of 10 ms (100 time steps per cycle) and element edge length of 0.5 mm was found to be sufficient as it resulted in <5% change in the time-averaged stress. The blood RBCs/plasma and WBCs were modeled as discrete elements [48] with diameters of 8 and 16 µm, respectively.

Material properties were derived from the experimental tests described in Section 2.1. Axial and circumferential Cauchy stress–strain curves of the ATA, DTA, and ATAA tissues were measured, and the initial and maximum elastic modulus were calculated. The linear elastic and nonlinear hyperelastic (5-parameter Mooney–Rivlin) mechanical properties of the tissues were calculated and listed in Table 1 and Table 2, respectively. The circumferential hyperelastic properties were used for the healthy and diseased FE models. The mechanical properties of the aortic arch were assumed the same as the ATAs. A 5-parameter Mooney–Rivlin isotropic hyperelastic constitutive equation used to address the nonlinear stress–strain relationship of the tissues [49,50]. In an isotropic hyperelastic material, the strain energy density function, *W*, is a scalar function of the right Cauchy–Green deformation tensor, *C*. The scalar function is composed of either the principal invariants or the principal stretches of the deformation, both of which are derived from the right Cauchy–Green stretch tensor [51]. Assuming that the aorta is nearly incompressible [52,53], we use the same strain energy density function as in Refs. [54,55], of Mooney–Rivlin type, which may be written as such [56]:

(1) $$\begin{array}{ccc} W=C_{10}\left({\bar{I}}_{1}-3\right) & + & C_{01}\left({\bar{I}}_{2}-3\right)+C_{20}{\left({\bar{I}}_{1}-3\right)}^{2}+C_{11}\left({\bar{I}}_{1}-3\right)\left({\bar{I}}_{2}-3\right)\\ & + & C_{02}{\left({\bar{I}}_{2}-3\right)}^{2} \end{array}$$

where J=det(F) and F is the deformation gradient. ${\bar{I}}_{1}$ and ${\bar{I}}_{2}$ are the first and second invariants of the left Cauchy–Green strain tensor, B, respectively. For a normalized deformation gradient $\bar{F}=J^{-\frac{1}{3}}\bar{F}$, the left Cauchy–Green strain tensor is: $B=\bar{F}{\bar{F}}^{T}$. The material coefficients ($C_{ij}$) were obtained from the experimental results through the nonlinear least square optimization method described in our prior publications [39,40,50,57,58,59]. The hyperelastic material coefficients for ATA, DTA, and ATAA tissues are provided in Table 2.

### 2.4. Red Blood Cell, White Blood Cell, and Plasma Modeling Using Discrete Elements

A set of discrete elements were defined in the TA lumen to represent the RBCs, WBCs, and plasma in the fluid domain. The blood in the healthy ATA/DTA simulations comprised 7374 particles, representing RBCs (3319 ~ 45%), WBCs (51 ~ 0.70%), and plasma (4004 ~ 54.30%). The blood in the ATAA simulations comprised 8668 particles, representing RBCs (3900 ~ 45%), WBCs (62 ~ 0.70%), and plasma (4706 ~ 54.30%). These numbers were chosen through a set of molecular weight pre-simulations (data are not reported here). Each group of discrete elements had its own physical properties and, due to blood incompressibility, discrete elements kept their inter-distance constant. Discrete elements are two-node elements with a force update that may be written as follows [60]:

(2) $${\hat{f}}^{i+1}={\hat{f}}^{i}+\Delta \hat{f}$$

where the superposition *i* + 1 indicates the time increment and the superposed caret indicates the force in the local element coordinates, i.e., along the axis of the element. In the default discrete element case, no orientation vector is used, so the global components of the discrete element force are obtained by using the element’s direction cosines:

(3) $$\left\{\begin{array}{c} F_{x}\\ F_{y}\\ F_{z} \end{array}\right\}=\frac{\hat{f}}{l}\left\{\begin{array}{c} \Delta l_{x}\\ \Delta l_{y}\\ \Delta l_{z} \end{array}\right\}=\hat{f}\left\{\begin{array}{c} n_{x}\\ n_{y}\\ n_{z} \end{array}\right\}=\hat{f}n$$

where

(4) $$\Delta l=\left\{\begin{array}{c} \Delta l_{x}\\ \Delta l_{y}\\ \Delta l_{z} \end{array}\right\}=\left\{\begin{array}{c} x_{2}-x_{1}\\ y_{2}-y_{1}\\ z_{2}-z_{1} \end{array}\right\}$$

*l* is the length:

(5) $$l=\sqrt{\Delta l_{x}{}^{2}+\Delta l_{y}{}^{2}+\Delta l_{z}{}^{2}}$$

And $\left(x_{i},y_{i},z_{i}\right)$ are the global coordinates of the nodes of the spring element. The forces in Equation (3) are added to the first node and subtracted from the second one. For a node tied to the ground, we used the same approach, but, for the $\left(x_{2},y_{2},z_{3}\right)$ coordinates in Equation (2), the initial coordinates of node 1 (i.e., $\left(x_{0},y_{0},z_{0}\right))$ are used instead; thus:

(6) $$\left\{\begin{array}{c} F_{x}\\ F_{y}\\ F_{z} \end{array}\right\}=\frac{\hat{f}}{l}\left\{\begin{array}{c} x_{0}-x_{1}\\ y_{0}-y_{1}\\ z_{0}-z_{1} \end{array}\right\}=\hat{f}\left\{\begin{array}{c} n_{x}\\ n_{y}\\ n_{z} \end{array}\right\}$$

The increment in the element force is determined from the user-specified force–displacement relation with linear elastic relationship between the force–displacement/velocity. An orientation vector for a discrete element is defined as follows:

(7) $$m=\left\{\begin{array}{c} m_{1}\\ m_{2}\\ m_{3} \end{array}\right\}$$

This vector is defined to control the direction the spring acts. We considered the portion of the displacement that lies in the direction of the vector. The displacement of the spring is updated based on the change of length given by:

(8) $$\Delta l=l-l_{0}$$

where $l_{0}$ is the initial length in the direction of the vector and l is the current length given for a node to node spring by:

(9) $$l=m_{1}\left(x_{2}-x_{1}\right)+m_{2}\left(y_{2}-y_{1}\right)+m_{3}\left(z_{2}-z_{1}\right)$$

and for a node to ground spring by:

(10) $$l=m_{1}\left(x_{0}-x_{1}\right)+m_{2}\left(y_{0}-y_{1}\right)+m_{3}\left(z_{0}-z_{1}\right)$$

The nodal forces are then given by:

(11) $$\left\{\begin{array}{c} F_{x}\\ F_{y}\\ F_{z} \end{array}\right\}=\hat{f}\left\{\begin{array}{c} m_{1}\\ m_{2}\\ m_{3} \end{array}\right\}$$

The orientation vector can be either permanently fixed in space or acting in a direction determined by two moving nodes, which must not be coincident but may be independent of the nodes of the spring. In the latter case, we recomputed the direction in every cycle according to:

(12) $$\left\{\begin{array}{c} m_{1}\\ m_{2}\\ m_{3} \end{array}\right\}=\frac{1}{l^{n}}\left\{\begin{array}{c} x_{2}^{n}-x_{1}^{n}\\ y_{2}^{n}-y_{1}^{n}\\ z_{2}^{n}-z_{1}^{n} \end{array}\right\}$$

In Equation (10), the superscript, *n*, refers to the orientation nodes. For the case where we consider motion in the plane perpendicular to the orientation vector, we consider only the displacements in the plane, $\Delta l^{p}$, given by:

(13) $$\Delta l^{p}=\Delta l-m\left(m.\Delta l\right)$$

The displacement of the spring is updated based on the change in length in the plane given by:

(14) $$\Delta l^{2}=l^{p}-l_{0}^{p}$$

where $l_{0}^{p}$ is the initial length in the direction of the vector and l is the current length given for a node to node spring by:

(15) $$l^{p}=m_{1}^{p}\left(x_{2}-x_{1}\right)+m_{2}^{p}\left(y_{2}-y_{1}\right)+m_{3}^{p}\left(z_{2}-z_{1}\right)$$

and for a node to ground spring by:

(16) $$l^{p}=m_{1}^{p}\left(x_{0}-x_{1}\right)+m_{2}^{p}\left(y_{0}-y_{1}\right)+m_{3}^{p}\left(z_{0}-z_{1}\right)$$

where

(17) $$\left\{\begin{array}{c} m_{1}^{p}\\ m_{2}^{p}\\ m_{3}^{p} \end{array}\right\}=\frac{1}{\sqrt{\Delta l_{x}^{p^{2}}+\Delta l_{y}^{p^{2}}+\Delta l_{z}^{p^{2}}}}\left\{\begin{array}{c} \Delta l_{x}^{p}\\ \Delta l_{y}^{p}\\ \Delta l_{z}^{p} \end{array}\right\}$$

After computing the displacements, the nodal forces are then given by:

(18) $$\left\{\begin{array}{c} F_{x}\\ F_{y}\\ F_{z} \end{array}\right\}=\hat{f}\left\{\begin{array}{c} m_{1}^{p}\\ m_{2}^{p}\\ m_{3}^{p} \end{array}\right\}$$

To account for strain rate effects, we scaled the forces based on the relative velocities that apply to all springs. The forces are computed from the spring elements that are assumed to be the static values and scaled by an amplification factor to obtain the dynamic value:

(19) $$F_{dynamic}=\left(1+k_{d}\frac{V}{V_{0}}\right)F_{static}$$

where $k_{d}$ is a constant parameter depending on the stiffness of the spring, V is the absolute relative velocity, and $V_{0}$ is the dynamic test velocity. Herein, V=2.5 m/s, $k_{d}=1.5$, and $V_{0}=15\;m/s$.

The deflection limit in compression and tension is restricted in its application to no more than one spring per node subject to this limit, and no deformable bodies only. When the limiting deflection is reached, momentum conservation calculations are performed and a common acceleration is computed:

(20) $$a_{common}=\frac{{\hat{f}}_{1}+{\hat{f}}_{2}}{m_{1}+m_{2}}$$

The discrete elements in this study had linear elastic force–displacement relation as follows:

(21) $$\hat{f}=K\Delta l$$

where K is the element’s stiffness and Δl is the change in length of the element. The physical and material properties of the RBC, WBC, and plasma are reported in Table 3.

### 2.5. Computational Fluid Dynamics

The incompressible CFD (ICFD) solves the Navier–Stokes equations, the equations of motion and continuity [64]. The continuity equation in the differential form is:

(22) $$\frac{\partial \rho }{\partial t}+\nabla \cdot \left(\rho \vec{u}\right)=0$$

Herein, the *ρ* is constant as the blood is assumed an incompressible flow. Thus, a volume continuity equation can be simplified as:

(23) $$\nabla \cdot \vec{u}=0$$

The conservations of momentum and mass in an incompressible Newtonian fluid can be explained through the Navier–Stokes equations mixed with the continuity equations:

(24) $$\left(\frac{du_{i}}{dt}+u_{j}\frac{\partial u_{i}}{\partial x_{j}}\right)=\frac{\partial \sigma_{i.j}}{\partial x_{j}}+\rho f_{i}\;\;\mathrm{in}\;\;\;\Omega$$

(25) $$\frac{\partial u_{i}}{\partial x_{j}}=0\;\;\mathrm{in}\;\;\Omega \;\;\;\;$$

The stress tensor is represented by:

(26) $$\sigma_{ij}=-p\delta_{ij}+\mu \left(\frac{\partial u_{i}}{\partial x_{j}}+\frac{\partial u_{j}}{\partial x_{i}}-\frac{2}{3}\frac{\partial u_{i}}{\partial x_{i}}\delta_{ij}\right)$$

Since we assumed the blood is incompressible:

(27) $$\frac{\partial u_{i}}{\partial x_{i}}\ll \frac{\partial u_{i}}{\partial x_{j}}$$

Hence, Equation (27) can be represented as follows:

(28) $$\sigma_{ij}\approx -p\delta_{ij}+\mu \left(\frac{\partial u_{i}}{\partial x_{j}}+\frac{\partial u_{j}}{\partial x_{i}}\right)$$

Likewise, $\frac{\partial \sigma_{ij}}{\partial x_{j}}$ can be simplified as follows:

(29) $$\begin{array}{cc} \frac{\partial \sigma_{ij}}{\partial x_{j}} & =-\frac{\partial p}{\partial x_{j}}\delta_{ij}+\frac{\partial }{\partial x_{j}}\left[\mu \left(\frac{\partial u_{i}}{\partial x_{j}}+\frac{\partial u_{j}}{\partial x_{i}}\right)\right]\\ = & -\frac{\partial p}{\partial x_{j}}\delta_{ij}+\mu \frac{\partial }{\partial x_{j}}\left(\frac{\partial u_{i}}{\partial x_{j}}\right)+\mu \frac{\partial }{\partial x_{j}}\left(\frac{\partial u_{j}}{\partial x_{i}}\right)\\ = & -\frac{\partial p}{\partial x_{j}}\delta_{ij}+\mu \frac{\partial }{\partial x_{j}}\left(\frac{\partial u_{i}}{\partial x_{j}}\right)+\mu \frac{\partial }{\partial x_{j}}\left(\frac{\partial u_{j}}{\partial x_{i}}\right)\\ = & -\frac{\partial p}{\partial x_{j}}\delta_{ij}+\mu \frac{\partial }{\partial x_{j}}\left(\frac{\partial u_{i}}{\partial x_{j}}\right)+\mu \frac{\partial }{\partial x_{i}}\left(\frac{\partial u_{j}}{\partial x_{j}}\right)\\ \approx & -\frac{\partial p}{\partial x_{j}}\delta_{ij}+\mu \frac{\partial }{\partial x_{j}}\left(\frac{\partial u_{i}}{\partial x_{j}}\right) \end{array}$$

So, finally, we will have:

(30) $$\rho \left(\frac{\partial u_{i}}{\partial t}+u_{j}\frac{\partial u_{i}}{\partial x_{j}}\right)=-\frac{\partial p}{\partial x_{i}}+\mu \frac{\partial^{2}}{\partial x_{j}\partial x_{i}}+\rho f_{i}\;\;\;\mathrm{in}\;\;\Omega \;\;\;$$

(31) $$\frac{\partial u_{i}}{\partial x_{i}}=0\;\mathrm{in}\;\;\Omega \;$$

The 10-noded tetrahedral element type was used to mesh the fluid domain. The flow boundary was treated as a rigid incompressible CFD surface, where no-slip boundary conditions were applied. The blood was modeled to be homogeneous, Newtonian, and viscous, with the density and dynamic viscosity of 1059 kg/m^3^ and 3.5 mPa·s [65], respectively. The velocity boundary was imposed to the model as, over the flow boundary, the direction of the flow is being determined by the momentum equations at each load increment. Since a no-slip boundary condition was applied on the flow boundary, the normal and tangential velocities must vanish, which can be found in the following equations:

(32) $$u_{i}={\vec{v}}_{i}\;\;\;\;\mathrm{on}\;\;\;\;\;\;\Gamma_{v}$$

where ${\vec{v}}_{i}$ represents the velocity function that was imposed on the boundary.

The stress tangential to the surface was vanished, and the stress normal to the flow boundary was balanced with any sort of externally applied normal stresses as follows:

(33) $$t_{i}=\sigma_{ij}n_{j}={\vec{t}}_{i}\;\;\;\;\;\mathrm{on}\;\;\;\;\Gamma_{f}$$

where $n_{j}$ is the direction cosine of the outward normal on the boundary with respect to the $x_{j}$ axis. Both surfaces $\Gamma_{v}$ and $\Gamma_{f}$ are two disjoint non-overlapping subsets of the boundary Γ. The velocity and pressure at the initial time were set for the Lagrangian Navier–Stokes problem as follows:

(34) $$v_{i}\left(x_{i},0\right)=v_{i}^{0}\left(x_{i}\right)$$

(35) $$p\left(x_{i},0\right)=p^{0}\left(x_{i}\right)$$

where the initial velocity $v_{i}^{0}$ has to satisfy the incompressibility constraint $\frac{\partial v_{i}}{\partial x_{i}}=0$.

**Figure 3:**
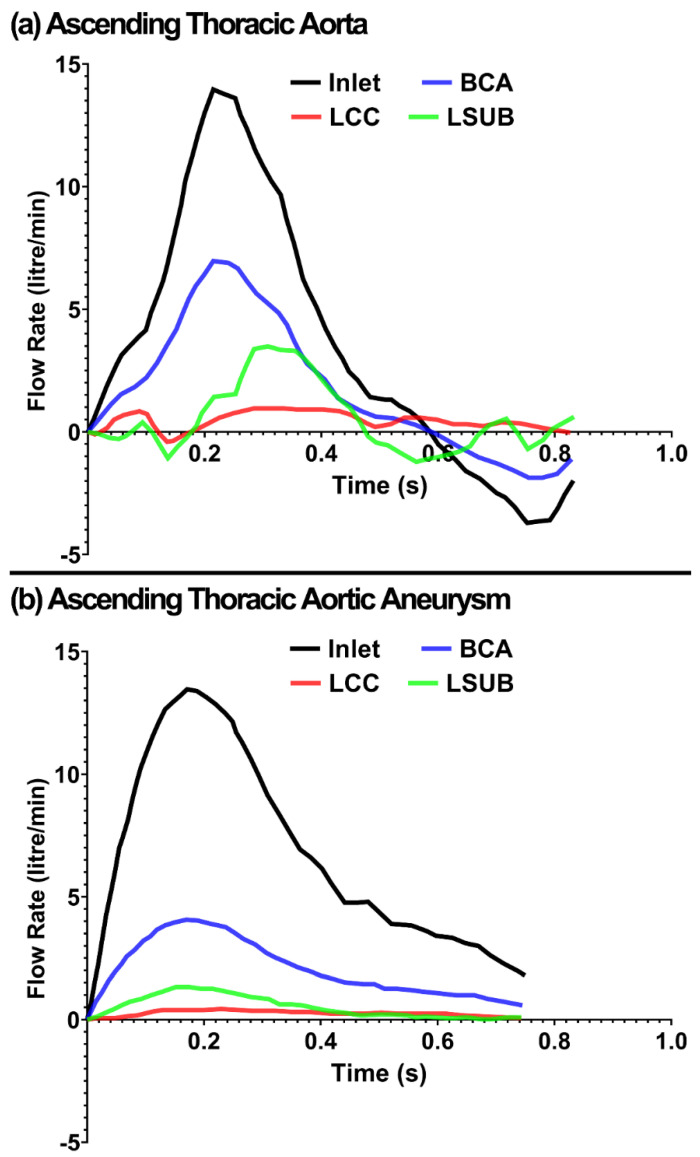
The inlet flow rate waveform in the (**a**) TA and (**b**) ATAA [31].

The simulations for the TA and ATAA were conducted by flow rate in the inlet and the apico-aortic branches (BCA, LCC, LSUB) [31], as shown in Figure 3. A multiscale approach was implemented to describe the hemodynamics at the descending aorta outlet by coupling the 3D domain with a three-element Windkessel model, as shown in Figure 2. The three-element Windkessel model was used to represent the physiological blood pressure and adopted from literature for a healthy (case 1) and an aneurysmal (case 2) TA [31]. The three parameters, including the peripheral resistance (resistor, R), the aortic compliance (capacitor, C), and the characteristic impedance (Z), were 1.36 × 10^7^ kg·m^−4^·s^−1^, 1.5 × 10^−8^·kg^−1^·m^4^·s^2^, and 2.28 × 10^−8^ kg·m^−4^·s^−1^ for the healthy and 1.65 × 10^7^ kg·m^−4^·s^−1^, 1.48 × 10^−8^ kg^−1^·m^4^·s^2^, and 2.77 × 10^−8^ kg·m^−4^·s^−1^ for the aneurysmal tissue, respectively. Since there was no information for the outlet boundary of the healthy and aneurysmal models in Ref. [31], the outlet pressures from the CFD simulations were used as the outlet pressure boundary for the FSI simulations. The CFD simulation on average took 10 min in our workstation.

### 2.6. Fluid–Structure Interaction

Deformable fluid and solid domains can be explained through an arbitrary Lagrangian–Eulerian (ALE) approach wherein the fluid mesh is updated to follow the structure’s motion [66,67]. The “ALE incompressible” material was used with the element formulation of “one-point ALE multi-material group” to address the fluid properties [68]. Briefly, three domains were defined as spatial, material, and reference. The Eulerian reference was achieved through the coincidence of the spatial domain and the reference domain. The Lagrangian reference was obtained through the coincidence of the material domain and the reference domain. Since both the material and spatial domains with respect to the reference domain are in motion, the material time derivative of a physical property ∅ in the reference configuration can be defined as:

(36) $$\emptyset =\emptyset_{,t}+c\cdot \nabla \emptyset$$

where ∅ is the material time derivative, and $\emptyset_{,t}$ is the time derivative when the coordinates in the reference domain are fixed. The convective velocity, *c*, is defined as:

(37) $$c=v-v^{mesh}$$

where *v* is the fluid velocity and *v^mesh^* is the mesh velocity. In the Eulerian reference, the mesh velocity is zero (*v^mesh^* = 0), while, in the Lagrangian reference, *v^mesh^* = *v* and *c* = 0.

The Lagrangian formulations were used to solve the solid TA wall problem that displacement of the nodes and the elements on a Lagrangian mesh correspond to the movements of blood, including the RBCs, WBCs, and plasma (fluid). The edges of the ALE fluid mesh always coincide (node to node) with the edges of the solid elements. In the Cartesian coordinate system, the displacement of the solid *u* in a domain $\Omega_{S}$ is governed by:

(38) $$\rho_{s}\frac{\partial^{2}u_{i}}{\partial t^{2}}=\sigma_{i}\left(u\right){,}_{j}+\rho_{s}g_{i}$$

with the initial and boundary conditions of:

(39) $$u_{i}={\hat{u}}_{i}\;\mathrm{on}\;\delta \Omega_{DS}\times \left[0,T\right]$$

The mass and momentum conservation laws can be defined as:

(40) $$v_{i,j}=0\;\mathrm{in}\;\Omega_{F}\times {\left[0,T\right]}_{\;}$$

(41) $$\frac{\partial v_{i}}{\partial t}+\left(v_{j}-v_{j}^{mesh}\right)v_{i,j}-\frac{1}{\rho_{F}}\tau_{ij,j}=g_{i}\;\;\;\mathrm{in}\;\Omega_{F}\times \left[0,T\right]$$

where *v_i_* and *ρ_F_* are the flow velocity and density, respectively. The term $v_{j}^{mesh}$ indicates the velocity of the mesh. If $v_{j}^{mesh}=0$, the Eulerian formulation will be achieved as the convective velocity of the mesh is null. If $v_{j}^{mesh}=v_{j}$, the Lagrangian formulation will be achieved as the convective velocity is equivalent to the fluid velocity. The quantity $v_{j}-v_{j}^{mesh}$ is the relative velocity and the stress tensor $\tau_{ij}$, commonly defined by:

(42) $$\tau_{ij}=\mu_{F}\left(v_{i,j}+v_{j,i}\right)-p\delta_{ij}$$

where $\mu_{F}$ is the fluid dynamic viscosity.

The momentum equation is solved with the initial and boundary conditions as follows:

(43) $$v_{i}\left(0\right)=0\;\mathrm{in}\;\Omega_{F}$$

(44) $$v_{i}=\hat{v_{i}}\;\mathrm{on}\;{\delta \Omega }_{DF}\times \left[0,T\right]$$

where $\hat{v_{i}}$ is the imposed velocity components on ${\delta \Omega }_{DF}$.

The boundary conditions on the FSI surface ${\delta \Omega }_{I}$ are defined as:

(45) $$v_{i}=\frac{\partial u_{i}}{\partial t}\;\mathrm{on}\;{\delta \Omega }_{I}\times \left[0,T\right]$$

And *p =* 0 on the TA boundary [69].

This method allows coupling between the fluid and solid sets via ALE coupling nodal penalty [60]. The normal and tangential damping coefficients between the discreet elements were defined as 0.7 and 0.4, respectively [60]. Static and rolling coefficients were defined as 0.41 and 0.001, respectively [60]. The scale factor of normal spring constant was defined as 0.01. The shear factor between the discreet elements was 0.0029. The blood was modeled to be homogeneous, Newtonian, and viscous, with the density and dynamic viscosity of 1059 kg/m^3^ and 3.5 mPa·s [65], respectively.

The centerline of the aorta was reconstructed using a custom Matlab script, and velocity vectors aligned with the centerline were assigned at each cross-section of the aortic wall. The input flow rates (Figure 3) were applied normal to the aorta cross-section (Figure 2). The inlet, outlet, and three apico-aortic branches (BCA, LCC, and LSUB) were all fixed in X, Y, and Z directions (Figure 2). A 10-core Intel^®^ Xeon^®^ CPU W-2155@3.30 GHz computer with 256GB RAM was used to run the simulations in explicit-dynamic FSI LS-DYNA. The simulations on average took ~200 h to run on our workstation.

## 3. Results

### 3.1. Experimental Results

**Figure 4:**
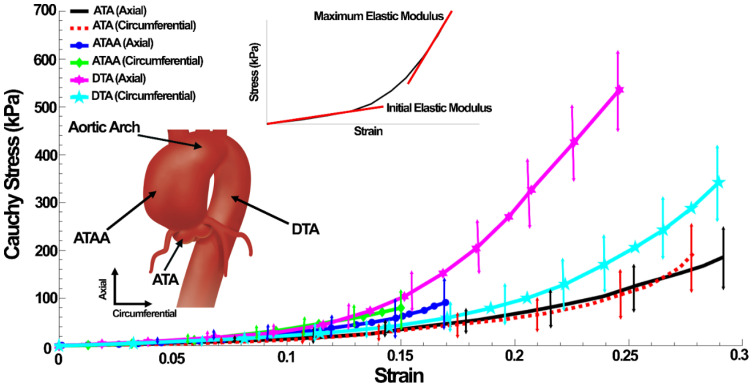
Cauchy stress–strain diagrams for the ATA, ATAA, and DTA in the axial and circumferential loadings. Schematic view of the aorta with locations where the aortic tissue was removed. Stress–strain curves and derivation of the initial and maximum elastic moduli.

The stress–strain curves in the ATA, ATAA, and DTA in both the axial and circumferential directions are shown in Figure 4. The linear mechanical properties of ATAs, DTAs, and ATAAs in the axial and circumferential loadings are reported in Table 1. The initial elastic modulus and failure strain in the axial direction were larger in the ATA but with smaller maximum elastic modulus and failure stress. The initial elastic modulus (126.19 ± 16.15 kPa, mean ± SD) of the ATAs in the axial direction was significantly larger (*n* = 9, *p* = 0.011 ≤ 0.05) than the circumferential (78.78 ± 9.55 kPa) direction (Table 1). However, regarding the maximum elastic modulus and failure stresses and strains, the differences were insignificant. The DTA showed significantly larger initial elastic modulus (254.74 ± 45.89 kPa) (*n* = 9, *p* = 0.006), maximum elastic modulus (1884.64 ± 169.14 kPa) (*n* = 9, *p* = 0.0017), and failure stress (535.33 ± 81.11 kPa) (*n* = 9, *p* = 0.035) in the axial direction compared to those of 188.95 ± 25.19, 1038.15±101.46, and 341.71 ± 71.69 kPa in the circumferential direction, respectively. However, the difference between the failure strains was insignificant. Comparison between the ATA and DTA revealed significantly larger initial elastic modulus (axial *p* = 0.0118 and circumferential *p* = 0.002), maximum elastic modulus (axial *p* = 0.0002 and circumferential *p* = 0.002), and failure stress (axial *p* = 0.002 and circumferential *p* = 0.03) for the DTA, while there was an insignificant difference in failure strain. Regardless of the loading directions, the DTA was found to be stiffer than the ATA and the differences in terms of extensibility were negligible. Regarding the ATAAs, the results revealed insignificantly larger initial elastic modulus (145.22 ± 20.85 kPa), maximum elastic modulus (506.64 ± 70.79 kPa), failure stress (90.68 ± 25.65 kPa), and failure strain (16.96 ± 5.79%) in the axial direction compared to those of 131.03 ± 22.58, 498.79 ± 65.98, 79.25 ± 32.14 kPa, and 15.00 ± 4.39% in the circumferential direction, respectively. The results also showed that the initial elastic moduli for the ATAA were significantly larger (axial *p =* 0.042 and circumferential *p =* 0.012) than that of the ATA. Regardless of the loading directions, maximum elastic modulus (axial *p =* 0.0007 and circumferential *p =* 0.029), failure stresses (axial *p =* 0.013 and circumferential *p =* 0.018), and strains (axial *p =* 0.014 and circumferential *p =* 0.039) showed significantly larger values for the ATA compared to the ATAA. Aneurysm also influenced the thicknesses and luminal diameters; the thickness (*n* = 9, *p =* 0.042) and diameter (*n* = 9, *p =* 0.005) in the ATA were significantly larger and smaller than that of the ATAA, respectively (Table 1). The hyperelastic material parameters are reported in Table 2.

### 3.2. Numerical Results

**Figure 5:**
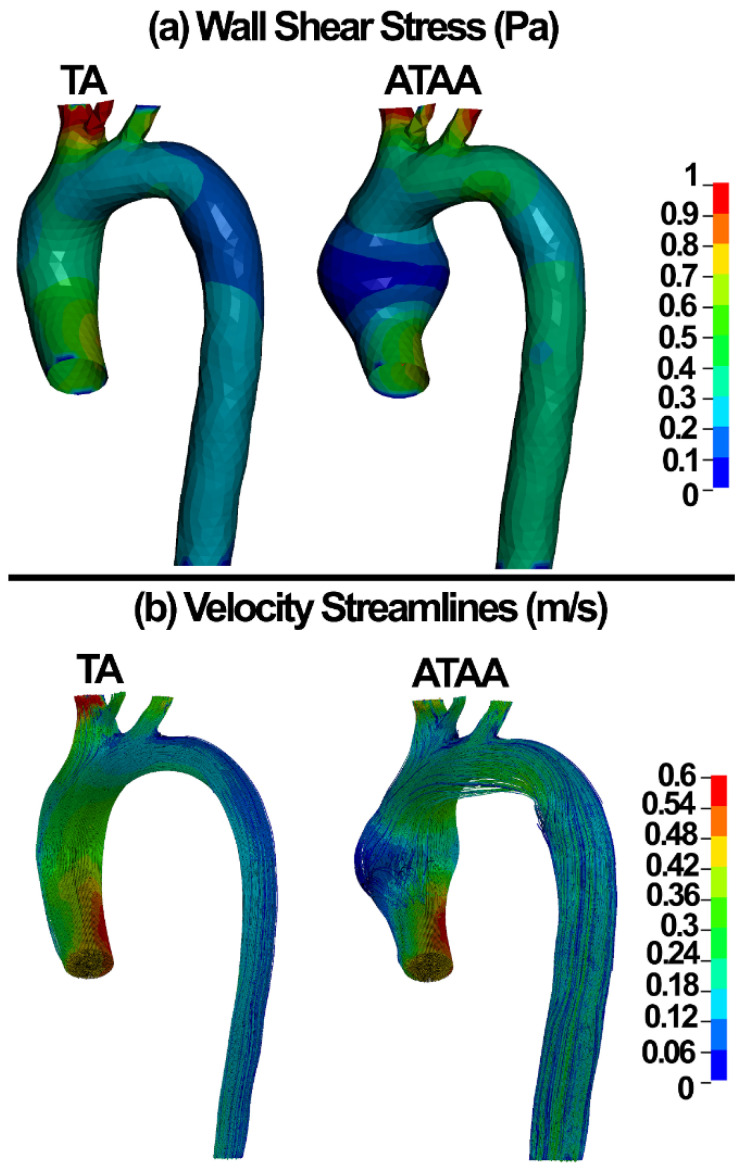
(**a**) Wall shear stress and (**b**) velocity streamlines in the ATA and ATAA.

The contour maps of wall shear stress and velocity streamlines in the healthy and aneurysmal aortic walls are shown in Figure 5.

**Figure 6:**
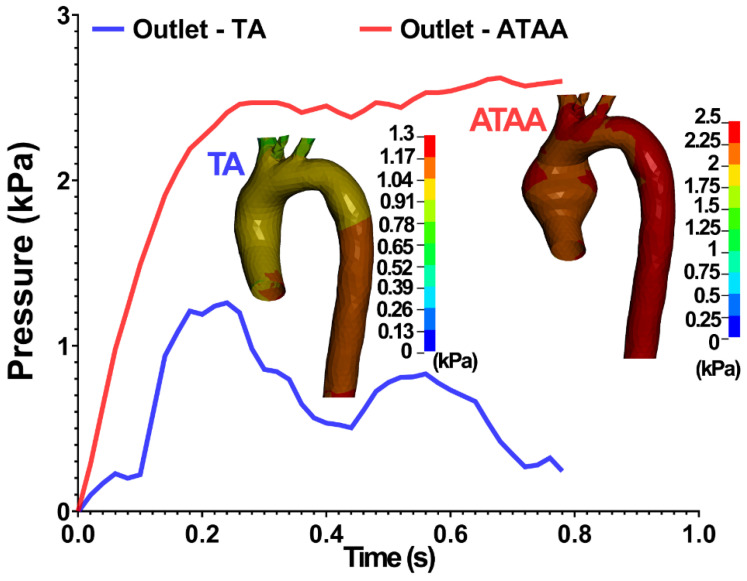
Outlet pressure boundary in the TA and ATAA from the CFD simulations. The flow pressure from the CFD simulations is also shown in the inset.

The pressure–time diagram in the output of the TA and ATAA using the CFD method are shown in Figure 6. The contours of fluid pressure in the TA and ATAA are also shown in the inset of the figure.

**Figure 7:**
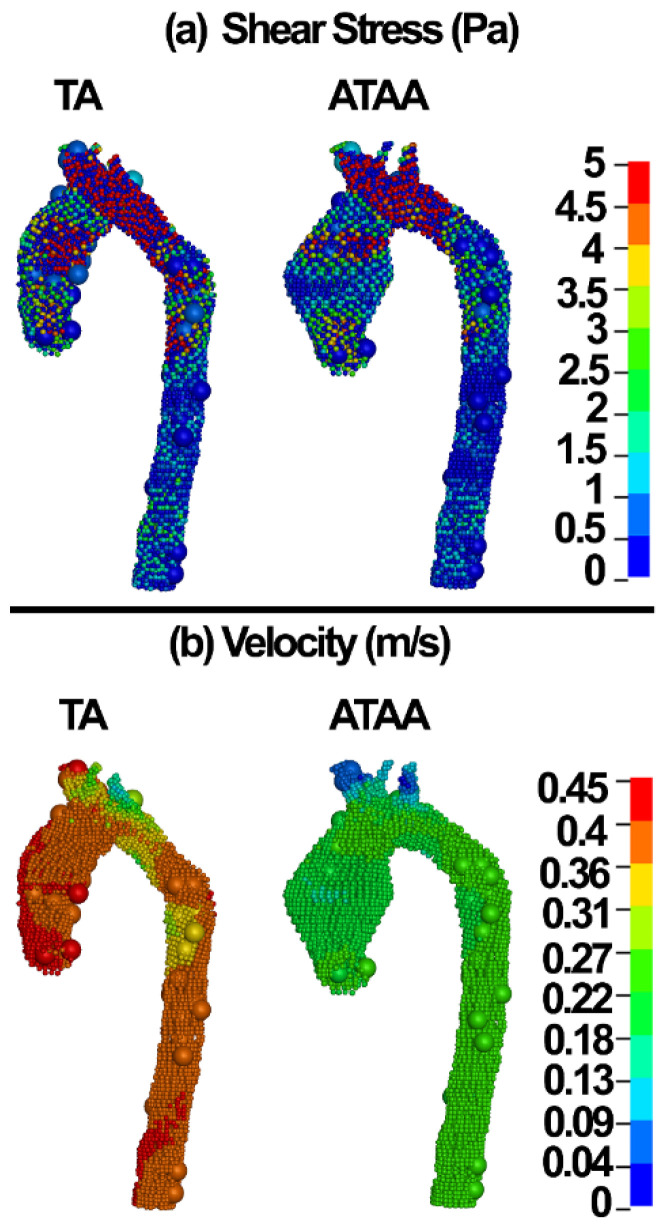
(**a**) Wall shear stress and (**b**) velocity in the blood components of the ATA and ATAA models.

The shear stress and velocity of the blood components in the TA and ATAA are shown in Figure 7.

**Figure 8:**
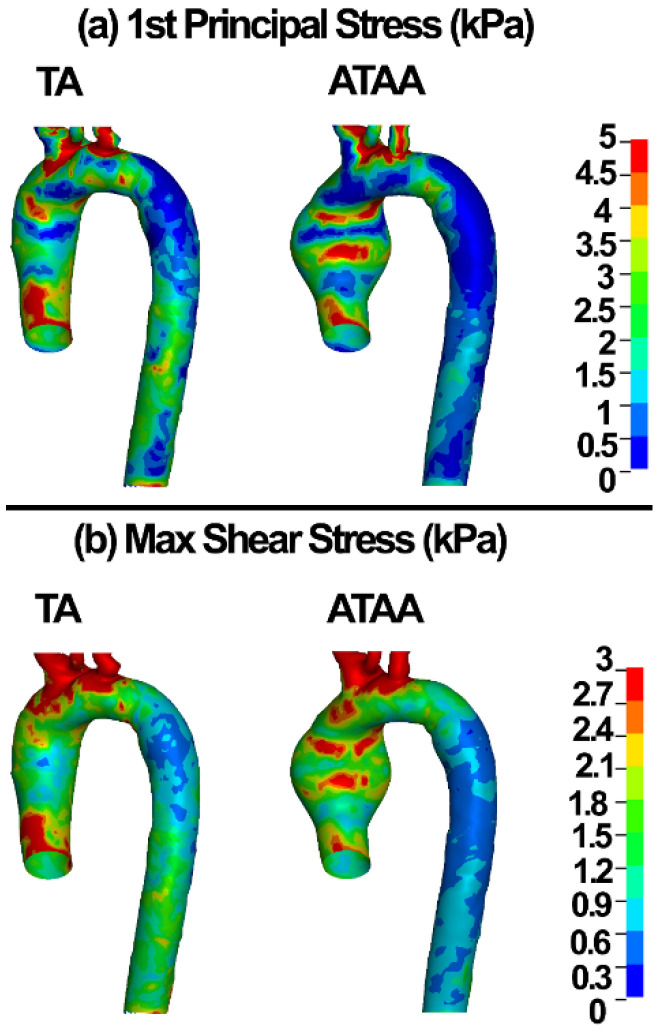
(**a**) 1st principal stress and (**b**) maximum shear stress in the ATA and ATAA walls.

The first principal and maximum shear stresses in the TA and ATAA walls are shown in Figure 8.

## 4. Discussion

This study is the first proof of concept for a complete FSI analysis in an ATAA using discrete elements to represent the blood components. The interactions between the blood components and the aortic wall were investigated to compute the stresses in the blood and the aortic wall.

Computational biomechanical modeling, such as the FE method, is ultimately aimed to assess the potential risk of rupture in the aneurysm. Biomechanical simulations permit accurate prediction of stresses and deformations in the aortic wall with various loading conditions, which is not achievable in vivo. Considering the interaction of the blood components, i.e., the RBCs, WBCs, and plasma, with the aortic wall, they could be instrumental for the rupture risk assessment, but only computational models can achieve such assessments. Usually, Eulerian or grid-based methods are used for blood flow simulations where quantities, i.e., densities, pressures, and velocities, are computed at fixed locations in space. However, particle-based approaches, namely discrete elements, enable to compute these quantities on particles that follow the fluid. This results in a substantial reduction in the simulation complexity compared to traditional FE approaches. The discrete elements method is interesting in numerical modeling of the blood flow due to free surface or interfacial flows for which no tracking or capturing algorithm is required, together with intrinsic kinematic and dynamic condition imposition [70,71]. In return, the computational costs are higher than mesh-based methods. This is because a large number of neighboring particles interact with each other compared to the small interaction distances of mesh-based methods. However, it should be noted that the accuracy of the discrete elements in blood simulations has been verified in different studies [70,71]. This seems very promising to further extend the applications of the discrete elements in the field. From the molecular point of view, it is important to understand how the components of the blood, such as RBCs, WBCs, and plasma, move inside the lumen of the healthy aorta and how this motion is altered in ATAAs. The discrete elements method permits such predictions.

The results revealed that the blood components experience smaller stresses in ATAAs compared to healthy TA (Figure 7a). This might be related to the larger diameter, which may be responsible for lower shear stresses (Figure 5a and Figure 7a) in the blood for similar flows as in healthy aortas.

It has been shown that the wall strength can vary significantly from one location to another in aneurysms [72]. We used different mechanical properties for ATA and DTA segments. Biomechanically, aneurysms rupture when the local stress exceeds the tissue strength. Therefore, it is important to noninvasively assess the wall stresses using patient-specific FE models. Tensile stress was mostly concentrated in the ATAA wall with 5 kPa (Figure 8a). The peak stresses were concentrated in the outer and inner curvature of the aneurysms right before the inlet cross-section (Figure 8). These regions have also been reported as the most prone to dissection due to suddenly increased blood pressure [16,28]. A wide range of wall stresses were reported for ATAAs as 471 ± 88 kPa [4], 242–535 kPa (circumferential direction), and 179–370 kPa (longitudinal direction) from diastole to systole [16], and 412–783 kPa [28].

The wall shear stress plays a major role in the generation, progression, and destabilization of atherosclerotic plaques [73]. It may have important implications on aneurysm dissections as well. Recent CFD analyses revealed the wall shear stress of 6.92 Pa in the pre-aneurismal and 8.53 Pa in the post-aneurismal sites of the aorta [74]. Condemi et al., found a maximum value of 6.69 Pa in the antero-lateral region of the aortic wall [75]. Mousavi and colleagues reported the wall shear stress and flow velocity of 1 Pa and ~0.5 m/s in the ATA and ATAA [31]. Herein, wall shear stress values of 1 and 5 Pa were computed in the ATAA models using the CFD and discrete elements-FSI methods, respectively (Figure 5 and Figure 7). The CFD velocity herein was in good agreement with Mousavi and colleagues [31]. The discrete element methods showed blood velocity values of 0.45 and 0.31 m/s in the ATA and ATAA, respectively, which is in good agreement with the CFD, which can be considered as approval of the application of the discrete element technique in ATAA rupture risk assessment.

It should be remembered that the present study is a first proof of concept for FSI analyses in aortic aneurysms using the discrete element technique. There remain a number of limitations. The constitutive equations of the wall did not take into account the tissue anisotropy. The geometry of the aneurysmal aorta was obtained by mere dilatation of the reference aortic geometry. Furthermore, the aortic geometry was assumed to be stress-free before the interactions with the fluid, without using an algorithm to find the zero pressure algorithm. The number of particles used in the simulations was much smaller than the number of RBC or WBC that can be found in the aorta, meaning that each particle only models the blood components averagely and not individually in our simulations. In addition, the results of the discrete element technique were not validated against experimental data. Eventually, only one case was simulated in healthy and aneurysmal situations. In the future, we plan to address all these limitations as the first results shown in this paper seem very promising.

## 5. Conclusions

This study is the first complete FSI analysis for an ATAA using the discrete element technique. FE models of healthy TA and ATAAs were established according to MRI datasets of healthy and diseased human individuals. The stresses in the wall of the aorta and components of the blood were computed and compared. The proposed discrete element approach could compute the stresses in the components of the blood in interaction with the healthy and diseased aortic wall. Future work will aim at developing a new rupture risk assessment for clinicians considering the interactions of the blood components and the aortic wall.

## Figures and Tables

**Table 1:** Axial and circumferential material properties of ATAs, DTAs, and ATAAs. Data are reported as mean ± SD.

Tissue	Initial Elastic Modulus (kPa)	Maximum Elastic Modulus (kPa)	Failure Stress (kPa)	Failure Strain (%)	Thickness (mm)	Lumen Diameter (mm)
ATA (Axial)	126.19 ± 16.15	591.08 ± 58.29	185.79 ± 28.14	29.16 ± 6.59	2.31 ± 0.31	28.14 ± 5.12
ATA (Circumferential)	78.78 ± 9.55	600.44 ± 79.16	194.79 ± 41.98	27.86 ± 6.28		
DTA (Axial)	254.74 ± 45.89	1884.64 ± 169.14	535.33 ± 81.11	24.59 ± 9.21	2.25 ± 0.19	29.49 ± 6.08
DTA (Circumferential)	188.95 ± 25.19	1038.15 ± 101.46	341.71 ± 71.69	28.95 ± 8.87		
ATAA (Axial)	145.22 ± 20.85	506.64 ± 70.79	90.68 ± 25.65	16.96 ± 5.79	1.91 ± 0.21	52.14 ± 6.88
ATAA (Circumferential)	131.03 ± 22.58	498.79 ± 65.98	79.25 ± 32.14	15.00 ± 4.39		

**Table 2:** Mooney–Rivlin parameters (mean values) of ATAs, DTAs, and ATAAs identified from the axial and circumferential stress–strain curves.

Tissue	C_10_ (kPa)	C_01_	C_02_	C_20_	C_11_
ATA (Axial)	82.38	−67.50	−3633.55	−2176.87	5743.45
ATA (Circumferential)	−1244.49	1283.22	50,754.36	32,263.82	−79,953.43
DTA (Axial)	1129.80	−1110.20	−95,766.17	−61,503.26	153,585.58
DTA (Circumferential)	271.69	−251.54	−3754.28	−671.36	3981.76
ATAA (Axial)	−1500.43	1546.61	172,626.85	127,687.77	−295,095.53
ATAA (Circumferential)	−311.13	331.29	70,892.11	51,270.33	−119,772.94

**Table 3:** Physical and material properties of the blood components [61,62,63].

Blood Components	Volume in the Blood (%)	ρ (kg/m^3^)	Shear Modulus (Pa)	Failure Stress (Pa)	Failure Strain (%)
Red blood cell	45	1106	9	12	75
White blood cell	0.70	1080	2.5	18.90	75
Plasma	54.30	1000	20	0.003	28

## Data Availability

The raw/processed data required to reproduce these findings cannot be shared at this time as the data are part of an ongoing study.
